# Intoxication of dogs and cats with common stimulating, hallucinogenic and dissociative recreational drugs

**DOI:** 10.1016/j.vas.2023.100288

**Published:** 2023-01-31

**Authors:** Ena Oster, Nikola Čudina, Hrvoje Pavasović, Andreja Prevendar Crnić, Frane Božić, Charbel Fadel, Mario Giorgi

**Affiliations:** a Department of Pharmacology and Toxicology, Faculty of Veterinary Medicine, University of Zagreb, Croatia; b Department of Veterinary Medicine, University of Sassari, Italy; c Department of Veterinary Sciences, University of Pisa, Italy

**Keywords:** Cats, Dogs, Illicit drugs, Intoxication, Recreational drugs, Toxicokinetics

## Abstract

Pets can have accidental, intentional, or malicious exposure to illicit drugs. It is a growing concern over the last decade because there is an increase in usage of illicit drugs in humans and diagnosis is difficult. Owners are often not aware of exposure, or they are reluctant to admit possession of recreational drugs in the household due to potential legal consequences. In addition, illicit drugs sold on the black market are often adulterated with other substances resulting in non-specific clinical presentation and aggravation of symptoms. There are affordable onsite diagnostic tests on the market which could facilitate diagnosis of intoxication with illicit drugs, but they give a lot of false positive results due to low specificity of the tests. In this paper we gathered information about the most common recreational drugs such as amphetamines, methamphetamine, 3,4-methylenedioxy-methamphetamine (MDMA), phencyclidine (PCP), lysergic acid diethylamide (LSD), psilocybin mushrooms and cocaine in terms of toxicokinetic properties, mechanism of toxic action, clinical presentation and treatment in dogs and cats.

## Introduction

Intoxication of pets with recreational (or illicit) drugs is getting more common over the last decade. Exposure to illicit drugs can be accidental, intentional, or malicious. Also, due to the wandering trait and non-selective appetite, dogs are more susceptible to any type of poisoning, including poisoning with illicit drugs (Gupta, 2019).

The increased use of illicit substances by their owners is likely the main factor contributing to the higher incidence in illicit drug intoxications in dogs and cats with those substances. If the owners keep illicit drugs in the household, their pets may have access to them. According to the European Drug Report (2022) published by the European Monitoring Centre for Drug Addiction (EMCDDA), approximately 83.4 million or 29% of adults in the European Union (ages 15 to 64 years) are thought to have taken illicit drugs at some point. Out of these, 14.4 million adults are thought to have consumed cocaine, 10.6 million MDMA, and 8.9 million amphetamines. Good example of the rise of incidence in drug exposure due to an increase of the drug availability and popularity, although not within the scope of this review, is cannabis. In the period between 2009 and 2014, the proportion of calls related to dog exposure to cannabis placed to the American Animal Poison Control center (APCC) increased from 0.84% to 1.53% of all toxicant calls. The states where legislation concerning cannabis was less strict had greater odds for a call being related to cannabis poisoning which indicates that poisoning incidence is related to socioeconomic and legislative variables of an area (Howard-Azzeh et al., 2021).

Furthermore, police dogs are at risk because they come in contact with large quantities of high purity chemicals during training or search operations (Dumonceaux & Beasley, 1990; Kisseberth & Trammel, 1990). In case of ingestion of whole bags of drugs, these should be removed either surgically or endoscopically, with special caution not to rupture the bag.

Because of the illegal nature of illicit drugs, diagnosis can be rather difficult. However, veterinarians are becoming more familiar with these intoxications as nowadays illicit drugs are gaining more attention in the veterinary community. Owners are still reluctant to acknowledge use of illegal substances, which leads to inaccurate, false, or deceptive exposure information. Therefore, unlike pet owners, veterinarians are significantly more likely to make a call related to opioid poisoning towards a poison control centre due to the fear of legal consequences (Howard-Azzeh et al., 2020). Furthermore, illicit drugs are often adulterated i.e. combined or contaminated with other pharmacologically active substances, making the diagnosis even more difficult. Some pharmacologically active substances, often found as impurities, are more toxic to pets than humans, like caffeine and xylitol.

Private and public forensic laboratories have verified methods to detect illicit drugs in body fluids and tissues in humans. Based on the authors’ knowledge, some methods have been also validated for veterinary patients, but these tests are costly and time-consuming. Over-the-counter test kits have been developed for analysis of human urine. One study evaluated the accuracy of urine test kits for drug intoxications in dogs by confirming the results using gas chromatography/mass spectrophotometry and found that urine test kits correctly identified the presence of barbiturates, some opioids, benzodiazepines, and amphetamines/methamphetamine in the urine (Teitler, 2009). But they didn't have patients with a suspicion of phencyclidine or cocaine intoxication, nor did they find positives with urine test kits. Nevertheless, over-the-counter urine test kits are rapid and affordable. In Table 1 are shown useful information regarding false positives with urine test kits provided by the American Society for the Prevention of Cruelty to Animals, based on data for humans.

**Table 1:** List of potential urine false positive results.

Illicit drugs	False positive results due to
Amphetamines	Trazodone, phenylpropanolamine, ranitidine, chlorpromazine, doxepin, fluoxetine, selegiline, amantadine, aripiprazole, bupropion, pseudoephedrine, phenylephrine, and atomoxetine
Phencyclidine (PCP)	Tramadol, diphenhydramine, ketamine, lamotrigine, venlafaxine, dextromethorphan, doxylamine and ibuprofen
Lysergic acid diethylamide (LSD)	Amitriptyline, chlorpromazine, diltiazem, doxepin, fentanyl, fluoxetine, metoclopramide, trazodone, bupropion, buspirone, risperidone, sertraline, verapamil, and methylphenidate

Adapted from American Society for the Prevention of Cruelty to Animals (2023).

There is a small number of papers published regarding the intoxications with illicit drugs due to difficulties with diagnosis (some patients could be misdiagnosed) and ethics of animal toxicology studies. Most of the paper about toxicokinetics and toxicodynamics are published in the 20^th^ century. We tried to collect as many data as possible about intoxications of dogs and cats with stimulating, hallucinogenic and dissociative illicit drugs, including some case reports, to facilitate diagnosis to veterinary clinicians.

## Methodology

Electronic databases of published scientific literature were the main source for this review. PubMed, Scopus, and Google Scholar were searched for toxicokinetics and toxicodynamics of the previously mentioned drugs. Additional articles of interest were obtained through cross-referencing of published literature. The primary key terms used were “illicit drugs” “recreational drugs”, “toxicology”, “cats” and “dogs”, which resulted in 403 search hits. Papers not related to stimulating, hallucinogenic or dissociative drugs, intoxications of dogs and cats or the mechanisms of toxic actions were excluded, leaving 47 papers which met the criteria for inclusion. Only English language papers were taken into consideration.

### Amphetamines and methamphetamine

Amphetamines, generally, refer to a class of psychotropic drugs originally used in humans for treatment of attention deficit hyperactivity disorder (ADHD) and narcolepsy. Specifically, amphetamine refers to α-methylphenethylamine (Heal et al., 2013). In contrast to amphetamines which have clinical benefits and specified usage, methamphetamine is a psychostimulant with strong addiction potential and is the second most frequently abused drug in the world (Buchweitz et al., 2022). High availability of methamphetamine is caused by its low cost, simplicity of synthesis and highly addictive characteristics. In rare cases and places, methamphetamine is still used for the same clinical conditions as amphetamine (Pei & Zhang, 2014). Amphetamines are commonly abused because of their stimulatory, euphoric, anorectic, and empathogenic properties (Carvalho et al., 2012). Between 2005 and 2014, methamphetamine constituted 0.10% of total dog, and 0.07% of total cat drug exposures reported to the American Association of Poison Control Centers (Swirski et al., 2020).

### Toxicity

Since there is an increase in the use of amphetamines and methamphetamine, occurrence of accidental pet intoxications is becoming more common (Stern & Schell, 2018). Median lethal dose for oral consumption in dogs is in the range 9–27 mg/kg (Harris et al., 2022). Oral LD_50_ for methamphetamine in dogs is 10 mg/kg (Buchweitz et al., 2022; Zalis et al., 1967), however the drug is now being sold at a much higher purity than previous forms of 40% purity which could make the new forms drastically more toxic (Chomchai & Chomchai, 2015). Methamphetamine intoxication in pets lacks official data for determining the incidence, but individual methamphetamine poisonings have been reported, including two cases, each concerning three patients with successful outcomes and one death in both case reports (Buchweitz et al., 2022).

### Toxicokinetics

In humans, amphetamines have a high oral bioavailability, high volume of distribution (approximately 4 L/kg), and low affinity for binding to plasma proteins (below 20%). Also, half-life is between 6 and 12 h, and most of them are metabolized in the liver with a high percentage being excreted without transformation (Carvalho et al., 2012; Kraemer & Maurer, 2002). Peak concentration is achieved 1 to 3 h after ingestion (Bischoff, 2018). Oral bioavailability of methamphetamine in dogs is approximately 67% and it distributes to most of body tissues. In one case report, ratio of metabolites to the parent compound was much higher than the reported ratio in humans. This could be due to interspecies differences in cytochrome P450 enzymes causing the drug to be transformed more quickly (Buchweitz et al., 2022). Amphetamines can pass the blood-brain barrier and methamphetamine tends to reach the highest concentration in the brain, the most out of all related drugs (Bischoff, 2018).

### Mechanism of toxic action

Amphetamines show affinity for both α- and β- adrenergic receptors, therefore mediating the release of noradrenaline. Via adrenergic stimulation, amphetamines stimulate cerebral cortex, reticular activating system and the medullar respiratory centre. Amphetamines as well prevent catecholamine reuptake and metabolism (Gupta, 2019). Methamphetamine and amphetamines cause release of dopamine in the CNS, show serotonergic and glutamatergic activity, and overall stimulate the sympathetic nervous system (Pei & Zhang, 2014).

### Clinical signs and symptoms

In humans, following oral use, the effects usually start within 30 min and last for several hours (United Nations Office on Drugs & Crime, 2003). Clinical signs resemble those of cocaine intoxication and are difficult to tell apart (Gupta, 2019). Clinical signs of poisoning in both animals and humans include hyperactivity, aggression, hyperthermia, tremors, ataxia, tachycardia, hypertension, mydriasis, circling, rhabdomyolysis, heart, kidney and liver dysfunction, ischaemia, seizures, head bobbing, and death (Gupta, 2019; Pei & Zhang, 2014). Lesions in experimental dog models include subendocardial and epicardial haemorrhages, and myocardial necrosis (Bischoff, 2018).

## Ecstasy (MDMA, 3,4-methylenedioxy-methamphetamine)

MDMA (or Ecstasy/Molly) is a type of amphetamine. Because its properties are similar to those of amphetamines, we will only list the differences here. MDMA, like methamphetamine, has psychedelic/hallucinogenic effects, making it a popular choice as a party drug.

### Toxicity

The biggest problem of party drugs is that they are often laced with adulterants such as cocaine, methamphetamine, ketamine, cathinones (or “bath salts”) and caffeine which could aggravate clinical signs of intoxication. Caffeine is an especially important adulterant for veterinary medicine since dogs and cats are more sensitive to it than humans. Also, cathinones can cause aggression and epileptic seizures (Baumann et al., 2013).

### Mechanism of toxic action

MDMA has a more potent effect on the release of serotonin, while methamphetamine is a more potent releaser of dopamine and noradrenaline (Rothman et al., 2001). Increased release of serotonin is observed up to 5 h after intravenous injection (Nishisawa et al., 1999). A study by Kirkpatrick et al., in 2012 compared behavioural and physiological effects of methamphetamine and MDMA on eleven adult volunteers. They found that methamphetamine improved cognitive and psychomotor performance, while MDMA had a negative effect on them.

### Clinical signs and symptoms

In pets, clinical signs develop within 30 min to 2 h after ingestion (Hooser & Khan, 2012). MDMA causes a potentially life-threatening condition called serotonin syndrome. Mild serotonin syndrome signs are mydriasis, shivering, sweating and mild tachycardia. Moderate serotonin syndrome is characterised by altered mental status (agitation, disorientation, excitement), autonomic hyperactivity (rigidity, tachycardia, hyperthermia) and neuromuscular abnormalities. If not treated, clinical signs develop into delirium, hypertension with tachycardia, life-threatening hyperthermia, and muscle rigidity (Mohammad-Zadeh et al., 2008). In dogs, MDMA is proven to cause circling, depression, dilated pupils, hyperactivity, rapid breathing, and salivation (Frith et al., 1987). Table 2 shows the clinical symptoms of serotonin syndrome according to the severity of the symptoms.

**Table 2:** Overview of clinical signs associated with serotonin syndrome.

Mild serotonin syndrome symptoms	Moderate serotonin syndrome symptoms	Life-threatening symptoms
Mydriasis Shivering Sweating Mild tachycardia	Altered mental status (agitation, disorientation, excitement) Autonomic hyperactivity (rigidity, tachycardia, hyperthermia) Neuromuscular abnormalities (tremor, clonus, hyperreflexia)	Delirium Hypertension Tachycardia Hyperthermia Muscle rigidity

Adapted from Frith et al. (1987); Mohammad-Zadeh et al. (2008).

## Phencyclidine (PCP)

Phencyclidine (PCP) is a dissociative anaesthetic commonly used as recreational drug. It is a drug of choice for many drug manufactures due to its easy and cheap manufacturing in clandestine laboratories. It is available in many forms, most commonly as variously coloured tablets or foil-wrapped powder (Bertron et al., 2018). In humans, it can be used orally, injected intravenously, inhaled, or smoked. Common street names for PCP are the peace pill, angel dust, crystal joints, rocket fuel, sawgrass, zoom, the sheets, and elephant tranquilizer (Journey & Bentley, 2022). It has hallucinogenic, depressant, and stimulant properties. It has more than 60 analogues, the most famous one being ketamine, which has one-twentieth to one-tenth the potency of PCP (Volmer, 2013).

PCP led to the discovery of ketamine (a dissociative anaesthetic used today in veterinary medicine), as well as other illicit and designer drugs. Furthermore, PCP is a frequent additive of many illegal drugs sold today (Bertron et al., 2018), which can aggravate clinical signs of intoxication with these drugs.

### Toxicity

In one study in dogs, pronounced clinical symptoms were observed with the dose of 2.5–10 mg/kg PO, in addition the dose of 25 mg/kg PO was lethal for all six poisoned dogs (Kisseberth & Trammel, 1990), nonetheless first signs of intoxication can be seen at doses as low as 1 mg/kg. In cats, marked clinical symptoms were seen at doses from 1.1 to 12 mg/kg IV (Boren & Consroe, 1981; Volmer, 2013).

### Toxicokinetics

PCP is well distributed (V_d_ 6.2 L/kg) and, due to its high lipophilicity, it achieves high concentrations in the CNS and adipose tissue. In dogs, 68% of a single dose is metabolised in the liver, and rest is excreted unchanged by the kidneys. In cats, 88% of the dose is excreted unchanged by the kidneys, with elimination half-life of approximately 1 hour (Volmer, 2013).

### Mechanism of toxic action

PCP is a non-competitive antagonist to the N-methyl-d-aspartate (NMDA) receptors in the CNS. It blocks the uptake of dopamine and noradrenaline, leading to sympathomimetic effects such as hypertension, tachycardia, bronchodilation and agitation. It can also bind to acetylcholine receptors and gamma-aminobutyric acid (GABA) receptors and cause sedation, muscarinic, and nicotinic signs (Journey & Bentley, 2022).

### Clinical signs and symptoms

In humans, inhalation has a 2–5 min onset of action, whereas oral administration can take 15 to 60 min Bey and Patel (2007). Dogs that ingested PCP appear depressed at low doses and stimulated at high doses. Symptoms include muscular rigidity, risus-sardonicus facial expression, increased motor activity, head weaving, incoordination, hypersalivation, nystagmus, opisthotonos, tonic-clonic convulsions, aggression, hyperthermia, tachycardia, hypertension, seizures, and coma (Bischoff, 2018; Boren & Consroe, 1981). Diagnosis is based on history of exposure, symptoms, and laboratory tests. Reported changes in chemistry parameters include acidosis, hypoglycaemia, electrolyte imbalances, increased creatinine phosphokinase and aspartate transaminase (Kisseberth & Trammel, 1990).

## Lysergic acid diethylamide (LSD)

Lysergic acid diethylamide, often abbreviated as LSD, is a hallucinogenic semi-synthetic product of lysergic acid, a compound commonly found in *Claviceps purpurea,* a fungus that contaminates cereals and grains. It was first used clinically as an enhancer of psychotherapeutic treatments in humans. In the 20th century, LSD was one of the leading drugs used for recreational purposes, often associated with sub-cultural, political, and religious movements. Nowadays, LSD is still a major drug of abuse (Passie et al., 2008). LSD exists in four different stereoisomers (D-, l-, d-iso, and l-iso) out of which only d-LSD isomer has hallucinogenic properties (Volmer, 2013). It is an odourless, tasteless, and colourless powder which can be applied in different forms including blotter papers, sugar cubes and stamps (Rimsza & Moses, 2005). Due to LSD criminalization, legal clinical research in humans since the 1970′s is scarce, on the other hand new perspectives started to arise in brain research, cluster headache treatment and psychotherapy (Passie et al., 2008). Consequently, LSD research in animals is even sparser. Nevertheless, hallucinatory-like responses and two specific types of behaviours in cats under LSD were observed in a study by Jacobs et al. (1977) where such behaviours increased in frequency with an increase in LSD dose, which gives a base for an animal research model.

### Toxicity

In the LSD study on cats conducted by Jacobs et al. (1977), toxicity was not directly measured, but the observed changes to a lesser extent developed in cats with 2–2.5 µg/kg dose of LSD applied intraperitoneally, and greater changes in behaviour developed in all cats with an intraperitoneal dose of 50 µg/kg. LSD is considered a relatively safe drug for humans in a recreational setting, with adverse reactions usually being short-lived, self-limiting, and psychological in nature (Kopra et al., 2022). Out of 10,293 international LSD users which participated in an online survey between November 2016 and January 2017, 1.0% reported seeking emergency medical treatment (Kopra et al., 2022). Toxicity of LSD varies between species with the lowest LD_50_ being 0.3 mg/kg IV in rabbits. LD_50_ in rats is 16.5 mg/kg IV and in mice 46–60 mg/kg IV (Freedman, 1969; Passie et al., 2008). While these species die due to paralysis and respiratory failure, monkeys can withstand a dose of 1 mg/kg IV without any lasting somatic effects (Evarts, 1956; Passie et al., 2008). Teratogenic effects in mice, rats and hamsters were observed in doses up to 500 μg/kg SC (Idänpään-Heikkilä & Schoolar, 1969; Passie et al., 2008). However, no teratogenic effects have been observed in humans (Passie et al., 2008).

### Toxicokinetics

A study concerning pharmacokinetics in healthy human individuals carried out by Dolder et al. (2017) shows that maximum plasma concentration values of 1.3 (1.2–1.9) and 3.1 (2.6–4.0) ng/mL were reached 1.4 and 1.5 hour after the oral administration of 100 and 200 µg of LSD, respectively, with the plasma half-life of 2.6 h (2.2–3.4 h). Subjective drug effects lasted for a mean of 8.2 h for a 100 µg dose, and 11.6 h for a 200 µg dose. Tissue distribution studies in cats dosed with LSD showed that the highest concentrations were detected in the gallbladder and blood plasma, with lower concentrations being present in the lungs, liver, brain, digestive tract, spleen and muscles, and the lowest concentration in fat tissue (Axelrod et al., 1957; Passie et al., 2008).

### Mechanism of toxic action

LSD is a serotonergic hallucinogen. In humans, it binds to serotonin (5-HT1A, 5-HT2A, 5-HT2C), dopamine (D2) and α2 adrenergic receptors. In addition, LSD binds less potently to α1 adrenergic, D1 and D3 receptors (Rickli et al., 2015, 2016; Simmler et al., 2016). In rats and mice, LSD additionally activates trace amine-associated receptor 1 (Rickli et al., 2016; Simmler et al., 2016).

Psychoactive properties of LSD in humans arise from its complex pharmacodynamics, which are not fully understood, however a huge contributing factor may be an increased glutamate release in the cerebral cortex, resulting in excitation (Nichols, 2004).

### Clinical signs and symptoms

Clinical signs in animals are not fully documented, but include mydriasis, sedation, depression, excitation, changes in behaviour and sometimes hallucinations. Such clinical signs usually appear within 90 min after exposure and last up to 12 h (Volmer, 2013).

Clinical signs observed in cats with intraperitoneal administration of LSD included rubbing, treading, or kneading, vocalization, grooming, head and body shakes, limb flicking, abortive grooming, investigatory and play behaviour, and hallucination-like behaviour (Jacobs et al., 1977). In doses up to 100 μg/kg IV in cats and rats, additional observations included tachycardia, tachypnoea, hyperglycaemia, hypertonia, and hyperthermia which can be attributed to the central stimulation of the sympathetic system (Passie et al., 2008; Salmoiraghi et al., 1957).

## Psilocybin mushrooms

Widespread use of mushrooms which contain psilocybin, also known as ‘magic mushrooms’ is common since the 1970′s, but its use can be traced even to ancient cultures (Gry et al., 2009; Kopra et al., 2022). Subjective effects of consumption in humans include synaesthesia, increased emotional lability and changes in sense of self, time and space (Kopra et al., 2022; Nichols, 2016). Psilocin is a psilocybin dephosphorylated metabolite (Zhuk et al., 2015). In addition, mushrooms containing psilocybin include *Psilocybe, Panaeolus, Conocybe and Gymnopilus* genera. Both psilocin and psilocybin are heat sensitive and some mushrooms contain additional pharmacological substances, including serotonin and tryptophan (Puschner, 2018). Although psilocybin is similar to other classical psychedelics, such as LSD and ayahuasca, it is relatively safe in comparison with them and according to available evidence will cause no neurophysiological deficits, organ damages or addiction (Johnson et al., 2018; Kopra et al., 2022; Nichols, 2016).

### Toxicity

In humans, there are only three acknowledged deaths caused by psilocybin containing mushrooms (Kopra et al., 2022; Lim et al., 2012). Lethal dose of psilocybin in humans is approximately 6 g, which is 1000 times more than the 6 mg threshold dose (Gable, 2004; Kopra et al., 2022). In dogs, only one known case report has been published on hallucinogenic mushroom ingestion (Kirwan, 1990; Puschner, 2018). In experimental mice models, determined LD_50_ value was 293.07 ± 1.02 mg/kg for synthetic psilocin, while for doses between 180 and 250 mg/kg no toxic effects were observed. Application of pure psilocin has shown higher toxic effects in contrast to methanolic fungi extracts (Zhuk et al., 2015).

### Toxicokinetics

Psilocin is a product of dephosphorylation of the pro-drug psilocybin. The transformation occurs in different tissues, but higher activity has been found in the liver and the kidneys in rodents (Horita & Weber, 1961; Puschner, 2018). It is generally accepted that the complete transformation into psilocin occurs before entering the systemic circulation (Puschner, 2018). Bioavailability of psilocin after oral administration of psilocybin in humans is 52.7 ± 20% and the maximum concentration is reached within 1.5 h due to fast absorption. Psilocin is eliminated mostly through urine in the form of psilocin-glucuronide, with some part excreted unchanged through bile (Hasler et al., 1997, 2002; Puschner, 2018).

### Mechanism of toxic action

As psilocybin turns into its active form psilocin whose effect on neurotransmitter systems is rather complex, most of its effects can be attributed to its structural similarity to serotonin which results in serotonin receptor stimulation (Mckenna et al., 1990; Puschner, 2018). Psilocin and similar hallucinogens behave as non-specific serotonin receptor agonists that bind to 5-HT1A, 5-HT2A and 5-HT2C receptors with different affinities. Some specific manifestations, such as head-twitch response in rats and mice, seem to arise due to the activation of 5-HT2A receptors (Matsumoto et al., 1997; Zhuk et al., 2015).

### Clinical signs and symptoms

In humans, exposure to normal doses (3–10 mg) of psilocybin result in tachypnea, tachycardia and systemic hypertension as acute effects (Carbonaro et al., 2018; Kopra et al., 2022). Overdoses with mushrooms lead to nausea, shivering, abdominal pain, and dizziness, while psychological effects in humans can result in clinical signs such as anxiety, disorientation, paranoia and panic attacks (Amsterdam et al. 2011; Kopra et al., 2022). Clinical signs which have been observed in dogs include ataxia, vocalization, overt aggression, increase in body temperature and nystagmus (Kirwan, 1990; Puschner, 2018). Although clinical signs usually occur within 0.5–1 hour, rarely they can be delayed up to 3 h after ingestion (Puschner & Wegenast, 2018).

## Cocaine

Cocaine is a natural alkaloid found in the leaves of the *Erythroxylon coca* plant and *E. monogynum* native to South America, and is commonly grown in Bolivia, Peru and Colombia (Queiroz-Neto et al., 2002). Cocaine is sold in salt form as a white powder, cocaine hydrochloride, with a purity of 12 to 60% (Kisseberth & Trammel, 1990). It is mixed with inert ingredients such as lactose, mannitol, starch or active components such as procaine, lidocaine, caffeine, amphetamines or PCP (Bischoff, 2018). Dogs and cats can get intoxicated by ingestion or inhalation. Between 2005 and 2014, cocaine constituted 0.05% of total dog, and 0.04% of total cat drug exposures reported to the American Association of Poison Control Centers (Swirski et al., 2020).

### Toxicity

LD_50_ in dogs is 3 mg/kg IV, and LD_99_ is 20 mg/kg IV. Dogs can tolerate 2–4 times the dose if the application is made PO (Bischoff, 2018). Drug sniffing police dogs can sometimes inhale cocaine during training or search operations.

### Toxicokinetics

Cocaine is lipid-soluble and is absorbed through all mucous membranes, including the nasal and oral cavity, digestive system, and alveoli. About 20% of the ingested dose is absorbed (Bischoff, 2018). It reaches the highest concentration in the plasma 15 min to 2 h after ingestion and passes the blood-brain barrier. Serum esterase and hepatic demethylation enzymes play a key role in cocaine metabolism (Volmer, 2013). Of the ingested/inhaled dose, about 20% is excreted unchanged in the urine (Kollias-Baker et al., 2003).

In humans, after oral ingestion, cocaine undergoes first-pass metabolism in the intestines and liver. It is absorbed from the gut and metabolized before reaching systemic circulation, leading to a wide difference in the pharmacokinetic behaviour between oral and IV dosing (Coe et al., 2018). But, after insufflation cocaine has effects and a pharmacokinetic behaviour qualitatively similar to those of IV cocaine, with an onset of action of 1–5 min, and a bioavailability of approximately 80% (Nelson et al., 2015). Furthermore, after insufflation or smoking, with a longer duration of action than an IV cocaine dose, these routes deliver a similar level of drug-appropriate response as the IV dose (Katz et al., 1991).

### Mechanism of toxic action

Cocaine increases the release of catecholamines and blocks the reuptake of noradrenaline, serotonin, and dopamine, which results in increased concentrations of these neurotransmitters at synapses (Queiroz-Neto et al., 2002; Vroegop et al., 2009). Effects on the heart are associated with intravenous administration (Kabas et al., 1990). Cocaine acts on the myocardium by blocking sodium ion channels, causing conduction disturbances, and lengthening of the R wave, increases calcium concentration in heart myocytes, which can lead to depolarization during systole and cause ventricular fibrillation. It leads to constriction of the coronary vessels, which results in hypoxia and infarction (Kabas et al., 1990).

### Clinical signs

In dogs exposed to cocaine, clinical signs typically develop rapidly, in around 10–15 min (Gwaltney-Brant, 2011). Clinical signs of cocaine poisoning manifest first as stimulation and then depression of the CNS (Kisseberth & Trammel, 1990). Hyperactivity, hyperaesthesia, tremors, and seizures occur in dogs. Other symptoms that appear in cocaine poisoning are ataxia, mydriasis, vomiting, hypersalivation, tremors, tachypnea, dyspnoea and acidosis. Pulmonary oedema, subendocardial and epicardial haemorrhages, degeneration of cardiac myofibrils, coronary vasoconstriction, pericardial effusion and lung haemorrhage can be seen on dissection (Bischoff, 2018). After intravenous applications, dogs show increased heart rate, cardiac output, and mean arterial pressure (Catravas & Waters, 1981). Frazier et al. (1998) described a high body temperature of 40.6 °C in the poisoning of one dog. In severe poisoning, hyperthermia and coma occur, and death occurs due to respiratory depression and heart failure.

## Diagnostics

As with all intoxications, diagnosis of intoxications with illicit drugs is a complex process which relies on anamnesis, clinical picture, pathomorphological signs and chemical and toxicological analyses. In some intoxications, mostly peracute ones, clinical signs could be absent which emphasizes the importance of a well taken anamnesis. Presence of a specific compound in a patient may not be a definitive proof that the exact compound is causing the clinical picture. Clinical signs for different intoxications may be overlapping which additionally complicates diagnostics (V. Srebočan & E. Srebočan, 2009). Consultation with a toxicologist is recommended before sample collection, and often the help of a toxicology laboratory for humans may be needed (Volmer, 2013).

Screening tests are available for many illicit drugs in diagnostic laboratories but are time-consuming and often times rather costly (Bischoff, 2018). As discussed before, amphetamines can be verified with use of chromatography and enzyme immunoassay, as well as with urine drug test kits (Teitler, 2009). They can be detected in blood, urine and saliva (Volmer, 2013). Diagnosis of PCP intoxication is also based on history of exposure, symptoms, and laboratory tests. In humans, clinical signs resemble those of overdoses with cocaine, amphetamines, anticholinergics and hallucinogens. Therefore, qualitative chromatographic or immunologic urine drug screening is used for confirmation of PCP intoxication as 9% of the active form is excreted by the kidneys. Use of fluorescence polarization immunoassay or high-pressure liquid chromatography may lead to false positive results for PCP if diphenhydramine, dextromethorphan or venlafaxine are present (Bey & Patel, 2007). Diagnosis of LSD intoxication in animals is mostly clinical due to difficulties with laboratory identification which are due to drug's fast metabolism (Volmer, 2013). A detailed medical history must be taken from the owner in order to identify the possibility of a contact with the drug. A study conducted by Dolder et al. (2015) showed efficacy in emergencies, by quantifying LSD and its main metabolite 2-oxo-3‑hydroxy LSD in serum and urine samples (Baquiran & al Khalili, 2022). Moreover, laboratory identification of LSD is possible with the help of immunoassays, thin-layer chromatography, high pressure liquid chromatography and liquid chromatography–mass spectrometry (Bischoff, 2012). Exposure of dogs to psilocybin can be demonstrated through detection of the psilocybin metabolite psilocin, or psilocin-glucuronide in urine and blood with high-performance liquid chromatography with column switching and electrochemical detection (Hasler et al., 2002). The diagnosis of cocaine poisoning is based on the owner's data on possible ingestion, clinical symptoms, as well as urine, blood (plasma) and stomach contents which are routinely tested in laboratories with the use of thin-layer chromatography, immunoassays, and gas chromatography/mass spectroscopy. The applicability of human urine tests has yet to be established in veterinary medicine (Bischoff, 2018).

## Treatment

Treatment of intoxications with stimulating, hallucinogenic and dissociative illicit drugs is often non-specific. Patients should be maintained in a dark, quiet, non-stimulating environment. The treatment is given until resolution of clinical signs.

### Gastrointestinal decontamination

If possible, the treatment should start with gastrointestinal decontamination. Depending on several factors, veterinary professionals can decide between emesis or gastric lavage. Some of these factors include physical and chemical properties of the ingested drug and, time elapsed from drug ingestion to presentation to the clinic and clinical signs of the patient. Unfortunately, rapid onset of clinical signs may preclude the use of emetics. Emesis in indicated within 4 h since toxin ingestion (Peterson, 2013). It is contraindicated in animals with high risk of aspirating vomitus, including patients with neurological depression and excitation, and some brachycephalic breeds (due to elongated soft palate). Also, emesis is contraindicated in patients that exhibit bradycardia because vomiting can cause sinus bradycardia due to vasovagal reflex initiated by distension of the upper oesophagus (Mehta et al. 1988). Central emetics used in veterinary medicine are apomorphine and xylazine. They act on the chemoreceptor trigger zone to induce emesis. Apomorphine is the emetic of choice in dogs because of its rapid onset and the ability to reverse its action with naloxone (Peterson, 2013). It is administered at a dose of 0.02 to 0.04 mg/kg by intravenous or intramuscular route. Xylazine is an emetic of choice in cats but with limited effectiveness at a dosage of 0.44 to 1 mg/kg by intramuscular or subcutaneous route (Peterson, 2013). Based on the authors’ knowledge, best peripheral emetics to use in dogs and cats are 3% hydrogen peroxide (1–3 mL/kg perorally) and sodium chloride solution (1 to 3 tablespoons in a cup of water perorally). Authors don't recommend using peripheral emetics because they are not as effective as central emetics and there is a higher possibility of vomitus aspiration (due to route of administration). Furthermore, sodium chloride solution can cause hypernatremia which can lead to cerebral oedema and convulsions. Gastric lavage is indicated if induction of emesis was contraindicated or ineffective and if the animal ingested large amount of drugs (dose close to LD_50_). Also, before the orogastric tube is removed, activated charcoal can be easily given through the tube. There are no papers published describing whole-bowel irrigation in intoxications of dogs and cats with illicit drugs.

In cocaine intoxication, gastrointestinal decontamination has a limited effect due to the rapid absorption of cocaine (Dumonceaux & Beasley, 1990). Police dogs that have ingested bags of cocaine should undergo careful endoscopy and surgical treatment to prevent rupture or obstruction. Gastrointestinal decontamination in LSD intoxication is not beneficial due to fast LSD absorption and self-limiting clinical signs (Volmer, 2013)

### Use of adsorbents

Adsorbents are non-specific antidotes that bind to toxins in the gastrointestinal tract until it is passed in the stool, thus reducing or preventing systemic adsorption of the toxins. Activated charcoal is effective at binding illicit drugs mentioned in this article, probably due to high lipid solubility of stimulating, hallucinogenic and dissociative illicit drugs. It should not be used in stuporous or comatose patients and in patients having seizures to avoid possible aspiration. The recommended dose for dogs and cats is 2–5 g/kg PO. In amphetamine intoxication, activated charcoal should be administered within 30 min after ingestion (Pei & Zhang, 2014). Unlike other illicit drugs listed in this article, in PCP intoxication, administration of activated charcoal should be repeated due to enterohepatic circulation of PCP (Volmer, 2013). Also, activated charcoal has been shown to increase the LD_50_ of PCP in dogs (Picchioni & Consroe, 1979).

### Ion trapping

Ion trapping inhibits the reabsorption of toxins across the renal tubular membranes by keeping the toxin in its ionised form in the urine where it can be excreted (Gupta, 2019). In patients intoxicated with PCP and amphetamines, ion trapping may boost drug elimination via kidneys (Gupta, 2019; Peterson, 2013). Ammonium chloride can be given at a dose of 100 mg/kg PO every 12 h (Peterson, 2013). Veterinary professionals are advised to monitor sodium and potassium levels, blood pressure and urine pH because urine acidification can alter acid-base balance. Also, as excretion rates of other drugs used to treat the patient can be altered, caution is advised.

### Intravenous lipid emulsion

Intravenous lipid emulsions (ILE) are used to treat toxicities with lipid soluble toxins, such as illicit drugs in this article. They form a “lipid sink” in serum which pulls lipid-soluble compound, resulting in a decrease of the free drug concentration available to tissues (Gupta, 2019; Lee, 2014; Volmer, 2013). The general dose recommended for dogs and cats is a bolus (1.5 mg/kg IV) followed by 8–15 ml/kg IV over 60 min (Hayes et al., 2016; Kormpou et al., 2018). In addition, a follow-up dose of 0.5 ml/kg/h IV (maximum 24 h) can be continued until clinical signs improve or serum is lipemic (Lee, 2014).

### Fluid therapy and forced diuresis

Fluid therapy is administered to aid in excretion of the drug (all illicit drugs listed in this article), to aid in perfusion (amphetamines, MDMA, PCP, cocaine, and psilocybin), and to prevent dehydration (Lee, 2014; Volmer, 2013). Balanced, isotonic crystalloid fluid can be given at 1.5 to 4 times a normal maintenance rate (Lee, 2014). To promote diuresis, diuretics can also be used, such as furosemide at a dose of 5 mg/kg every 6 to 8 h and mannitol at a dose of 1 to 2 g/kg IV every 6 h (Volmer, 2013). The possible side effects of forced diuresis include fluid overload and electrolyte abnormalities. Veterinary professionals are advised to monitor for signs of fluid overload (pulmonary and cerebral oedema) and electrolyte abnormalities (hyponatraemia, hypokalaemia).

### Supportive therapy

Since clinical signs of intoxication with stimulating, hallucinogenic and dissociative illicit drugs can vary and they depend on the dose of illicit drug ingested, every patient needs individual approach to supportive therapy. Symptomatic and supportive therapy should be administered, cardiac function, body temperature and acid-base status should be monitored.

Benzodiazepines, propofol and inhalation anaesthetics can be administered in case of severe hyperactivity, rigidity, or seizures. Diazepam is given at a dose of 0.5 mg/kg IV or 1 mg/kg rectal (Papich, 2016a). In mild clinical symptoms, midazolam can be given once as a bolus of 0.1–0.25 mg/kg IV. In severe case, constant rate infusion at the dose of 0.1–0.3 mg/kg/h IV can be given (Papich, 2016b). In amphetamine intoxication, administration of benzodiazepines may paradoxically exacerbate the neurological effects from amphetamine and are generally not recommended (Volmer, 2013). Phenothiazines are contraindicated because they may lower the seizure threshold and exacerbate the anticholinergic effect of drugs such as PCP (Puschner, 2018).

Body temperature should be closely monitored and controlled, since hyperthermia is associated with poor prognosis. In LSD intoxications, clinicians are advised to look for signs of rhabdomyolysis (Volmer, 2013).

Patients exhibiting mild signs of serotonin syndrome usually require only benzodiazepine treatment, if necessary. In case of more severe or life-threatening symptoms, cyproheptadine treatment is advised. Cyproheptadine is H1 antihistaminic that also blocks serotonin receptors. Furthermore, patients should be placed in intensive care unit, under sedation with cardiac monitoring, cooling measures, antihypertensive therapy (esmolol or nitroprusside), and mechanical ventilation if needed (Volmer, 2013).

In case of life-threatening tachyarrhythmias, e.g. in cocaine intoxication, β-blockers are given. Dose of propranolol in dogs and cats is 0.2–1 mg/kg PO every 8 h and 0.4–1.2 mg/kg PO every 8 h (Papich, 2016c), respectively. Caution is advised because β-blockers may cause systemic hypertension (Vroegop et al., 2009).

With prompt diagnosis, decontamination, monitoring and supportive care, prognosis is good (Volmer, 2013).

## Conclusion

Even though intoxications of dogs and cats with illicit drugs are anecdotally common, there is a lack of official reports that would support it. This is probably due to illegal nature of illicit drugs, difficult diagnosis, and owners’ incompliance. Diagnosis is even more difficult because clinical signs can significantly vary since almost all the illicit drugs have various toxicologically active adulterants and impurities.

Veterinary professionals will be held primarily accountable for the future growth toward a better diagnosis. It is critical to obtain an accurate history in order to gather information about the animal's environment, amount of exposure, time from exposure to onset of clinical signs, as well as type, severity, and duration of clinical signs, all while assuring clients that the information shared is confidential and the focus is to provide the best possible care for their pets. Veterinary clinics are also required to be connected to veterinary diagnostic laboratories that offer illicit drug screenings so that they can check various body fluids for the presence of illicit substances or their metabolites. The laboratories should be contacted as well for information on the types of samples needed and time required to complete the screens or tests. However, due to the short time frame of clinical signs, most animals will recover before the lab results are confirmed. Furthermore, readily available over-the-counter drug test kits can aid in the exclusion of a suspected case of illicit drug toxicosis and should be employed in all clinics. They can detect most commonly available illicit or recreational drugs such as amphetamines, cocaine, marijuana, opiates, and barbiturates by detecting drug metabolites in the urine. The kits are affordable, effective, and simple to use, however, their sensitivities and specificities may differ. In conclusion, veterinary professionals must be alert to possible illicit drug intoxications and aware that clinical signs could be non-specific due to various additives.

## Ethical statement

This manuscript is a review and does not need any ethical statement.

## Data availability statement

The data sets used and/or analysed during the current study are available from the corresponding author, upon request.

## Declaration of Competing Interest

The authors declare that they have no known competing financial interests or personal relationships that could have appeared to influence the work reported in this paper. No conflict of interest is present.
